# Fungal Cervical Abscess Complicated by Necrotizing Fasciitis Leading to Descending Necrotizing Mediastinitis: A Case Report

**DOI:** 10.7759/cureus.5369

**Published:** 2019-08-12

**Authors:** Zainab Abbasi, Hina Inam, Sajan Das, Sejal Neel, Saulat H Fatimi

**Affiliations:** 1 Internal Medicine, Liaquat University of Medical and Health Sciences, Jamshoro, PAK; 2 Cardiothoracic Surgery, The Aga Khan University, Karachi, PAK; 3 Emergency Medicine, Sindh Government Lyari General Hospital, Karachi, PAK; 4 Pediatrics, Sindh Government Children Hospital, Karachi, PAK

**Keywords:** fungal cervical abscess, necrotizing fasciitis, pneumomediastinum, internal jugular vein thrombosis, fungal cervical abscess

## Abstract

Cervical necrotizing fasciitis (CNF) is a rapidly spreading deep neck infection with a high mortality rate if left untreated. The occurrence of necrotizing infections in the head and neck region is uncommon; therefore, it is a rare cause of chest pain presenting to the emergency department. Here, we present an interesting case of fungal cervical skin abscess complicated by necrotizing fasciitis that progressed to involve the mediastinum, causing necrotizing mediastinitis with pneumomediastinum in an elderly female. The patient presented to the emergency department with chest pain, shortness of breath, and fever. She had a 10-day history of a mass in the anterior midline of her neck with odynophagia. After radiologic confirmation, she was taken to the operating room where she underwent incision and drainage with debridement and washout. Postoperatively, she was given broad-spectrum antibiotics empirically, which were later replaced with intravenous (I/V) fluconazole after culture reports. Prompt diagnosis and treatment lead to the early recovery of the patient and subsequent discharge without any complications. We report this case to draw the attention of emergency medicine physicians and clinicians to this rare and life-threatening but treatable condition. Expeditious diagnosis and treatment lead to early recovery and fewer postoperative complications.

## Introduction

Cervical necrotizing infections are a rapidly progressive and lethal variety of deep neck infections [1]. These infections are associated with greater mortality because of their proximity to various vital structures and possible extension into the mediastinum. Cervical necrotizing fasciitis (CNF) initially involves less vascularized tissues like fasciae and then spreads to surrounding tissues like muscles and fat tissue [2]. A fungal cervical abscess complicated by necrotizing fasciitis and then progressing caudally to involve the mediastinum, leading to a necrotizing infection of the mediastinum with the pneumomediastinum, which is an extremely rare presentation. Clinically, there are two forms of this disease: gaseous and purulent or a combination of both [2]. Surgical drainage with antibiotics or antifungals, depending upon the causative organism, are the mainstay of treatment [3]; however, most of these infections are still complicated due to a delay in diagnosis.

## Case presentation

**Figure 1:**
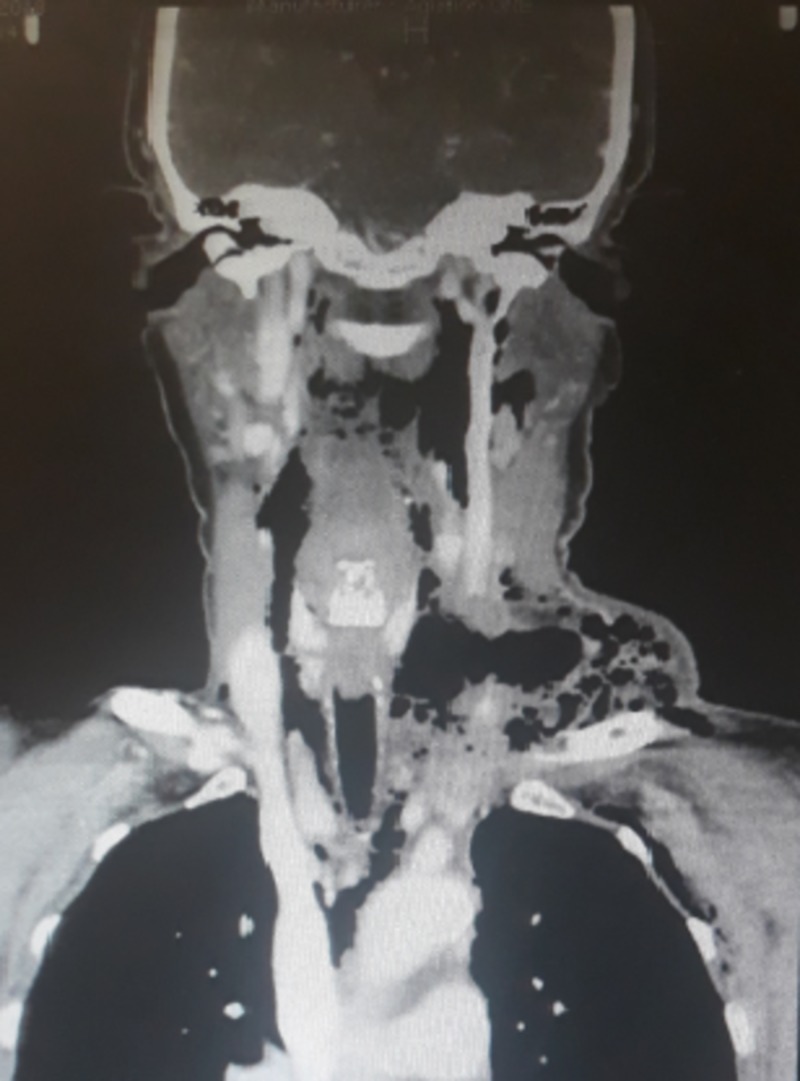
Coronal view of contrasted computed tomography scan of the neck and chest showing diffuse gas collection in the neck and left supraclavicular region extending into the mediastinum

**Figure 2:**
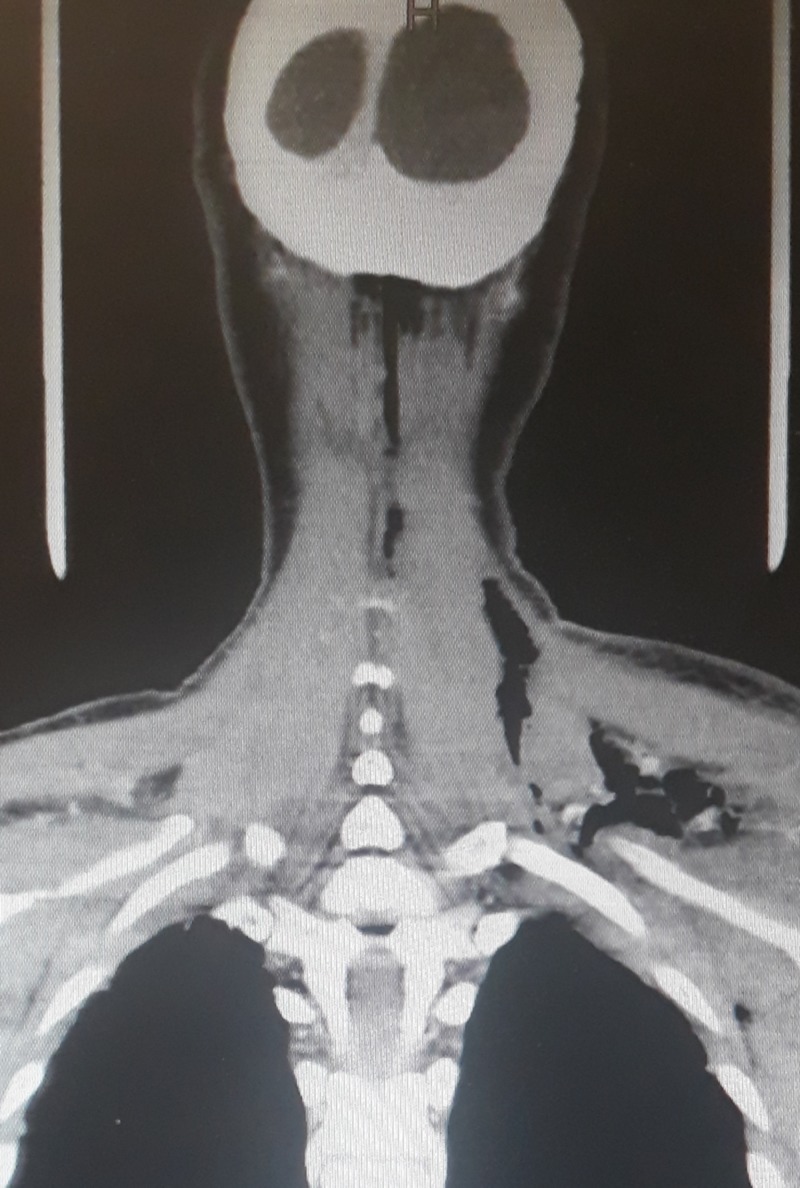
Coronal view of computed tomography of the neck and chest showing subcutaneous air extension into the left supraclavicular area

A 61-year-old woman, with no known comorbid conditions, presented to the emergency department of Aga Khan University Hospital (AKUH) with chest pain, shortness of breath, and fever. She had a 10-day history of a mass in the anterior midline of her neck with odynophagia and fever. On physical examination, she was found to have tachypnea, tachycardia, and bilateral basal crackles in both lungs. The patient also had subcutaneous emphysema and an erythematous anterior midline infrahyoid neck swelling, which was soft, tender, and fluctuant on palpation. Chest X-ray showed subcutaneous emphysema in the neck and axilla along with pneumomediastinum. Contrasted computed tomography (CT) scan neck and chest was ordered, which showed diffuse subcutaneous emphysema involving the deep veins of the neck, left supraclavicular region, and anterior and posterior chest wall, and left axilla (Figures 1-2).

**Figure 3:**
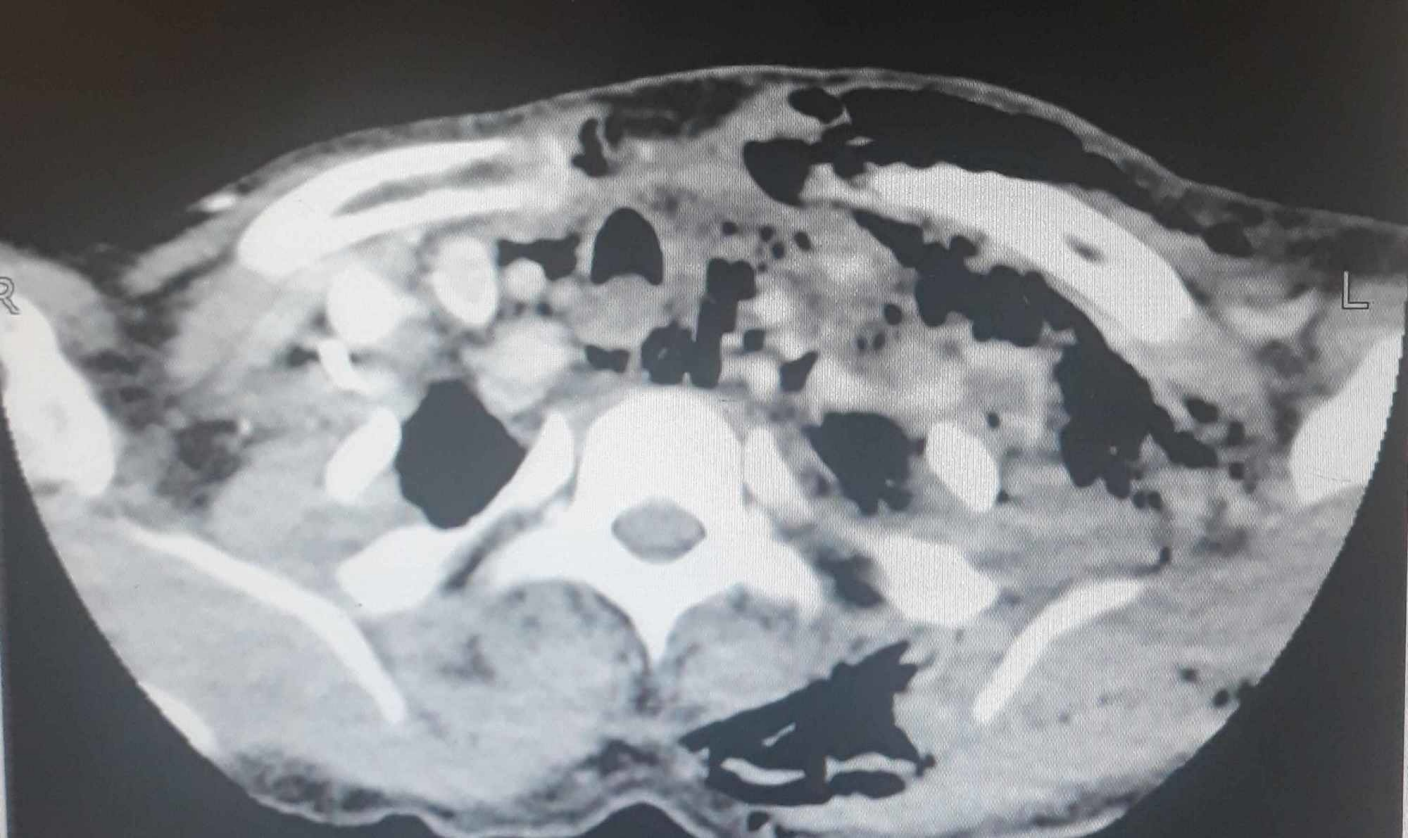
Contrasted computed tomography of the neck showing several multiloculated air pockets in the left paratracheal and prevertebral regions with a focal filling defect in the left internal jugular vein

Several multiloculated air pockets were seen in the left paratracheal and prevertebral regions. The largest air collection is seen in the left paratracheal region. A large fluid collection was seen tracking from the left infraglottic parapharyngeal space extending into the carotid space and involving the left supraclavicular fossa. This collection measured 10 cm in length. Numerous air specks were noted in this region. A focal filling defect within the left internal jugular vein representing thrombus was also appreciated along with an enlarged left-sided level II lymph node measuring 6 mm (Figure 3).

Based on the CT scan and clinical findings, a diagnosis of cervical necrotizing fasciitis with descending necrotizing mediastinitis was made, and surgical intervention was decided. The patient was taken to the operating room for transcervical drainage with superior mediastinotomy. Around 40 cc of pus mixed with air bubbles was drained, and debridement was done. Intraoperatively, the fasciitis was seen extending all the way to the axilla laterally, to the thoracic inlet medially, and up to the base of the mandible superiorly. Samples were sent for bacterial, fungal, and mycobacterial cultures. Postoperatively, the patient was started on broad-spectrum antibiotics empirically, which were later replaced with fluconazole after culture reports showed growth of Candida albicans sensitive to fluconazole. Daily wound dressing was also done regularly. The patient continued to improve clinically and was discharged on the fifth postoperative day.

## Discussion

CNF is a deep neck infection leading to 100% mortality if left untreated. It is a fast-spreading, soft-tissue infection that causes necrosis, starting with the tissues having less vascularity like the fasciae and gradually leads to the involvement of surrounding tissues like cervical fat and muscle [2].

The three layers of deep cervical fascia, namely, pretracheal, retrovisceral, and prevertebral/alar, divide the neck into three regions. Between the retrovisceral and prevertebral fascia, retropharyngeal and danger spaces are located, divided by a thin membrane of the alar fascia. The retropharyngeal is present along the entire length of the neck extending from the base of the skull superiorly to the level of carina in the posterior mediastinum inferiorly. It is situated behind the pharynx and the esophagus and anterior to the alar fascia [3].

The incidence of necrotizing mediastinal infections in patients with deep neck infections is 1.5%-3.6% [2-3]. Clinically, there are two types of CNF: suppurative and gaseous [4]. Our patient had fungal cervical necrotizing fasciitis leading to pneumomediastinum and internal jugular vein thrombosis, which is a very rare etiology that mostly occurs in immunocompromised patients. However, the patient was not in an immunocompromised state such as diabetes mellitus, obesity, alcoholism, renal/hepatic failure, or chronic corticosteroid intake. Other complications of descending necrotizing mediastinitis (DNM) include pneumonia, toxic shock, esophageal perforation, and pericardial effusion [5].

It has been reported that drainage and antibiotic therapy are insufficient in the management of DNM once the abscess has extended to a level below the tracheal bifurcation [6]. However, in our patient, drainage and debridement combined with systemic antifungal therapy were successful in eradicating the infection. The management of cervical necrotizing fasciitis with descending necrotizing mediastinitis includes airway management along with prompt surgical drainage and antibiotic/antifungal therapy [2,7]. Along with this, the primary source of infection or underlying systemic condition should be investigated to prevent recurrence [8]. Hyperbaric oxygen therapy is assumed to limit the magnitude and number of surgical debridement procedures, therefore reducing the mortality rate associated with necrotizing fasciitis, but we did not use hyperbaric oxygen because the published results remain inconclusive.

## Conclusions

Necrotizing mediastinitis should be suspected as a cause of chest pain presenting to the emergency department, especially in patients with cervical abscesses. Death is the most common sequelae due to a delay in proper diagnosis and the rarity of this condition. The present paper demonstrates that fungal cervical abscesses could also be complicated by necrotizing fasciitis, leading to descending necrotizing mediastinitis, which, if treated promptly with surgical debridement and systemic antifungal therapy, could lead to a substantial reduction in the mortality of this condition.

## References

[1] Lin C, Yeh FL, Lin JT, Ma H, Hwang CH, Shen BH (2001). Necrotizing fasciitis of the head and neck: an analysis of 47 cases. Plast Reconstr Surg.

[2] Karkas A, Chahine K, Schmerber S, Brichon PY, Righini CA (2010). Optimal treatment of cervical necrotizing fasciitis associated with descending necrotizing mediastinitis. Br J Surg.

[3] Panda NK, Mann SBS, Sharma SC (2000). Mediastinitis following deep neck infections: a therapeutic challenge. Indian J Otolaryngol Head Neck Surg.

[4] Gonlugur U, Guclu O, Karatag O, Mirici A, Derekoy S (2011). Cervical necrotizing fasciitis associated with descending necrotizing mediastinitis. Multidiscip Respir Med.

[5] Park H, Hickman N, Freund F, Skaryak LA, Fishel R (2010). Methicillin resistant Staphylococcus aureus infection in descending necrotizing mediastinitis from retropharyngeal perforation: a rare event. J Wound Ostomy Continence Nurs.

[6] Singhal P, Kejriwal N, Lin Z, Tsutsui R, Ullal R (2008). Optimal surgical management of descending necrotizing mediastinitis: our experience and review of literature. Heart Lung Circ.

[7] Deu-Martín M, Saez-Barba M, López Sanz I, Alcaraz Peñarrocha R, Romero Vielva L, Solé Montserrat J (2010). Mortality risk factors in descending necrotizing mediastinitis [Article in Spanish]. Arch Bronconeumol.

[8] Abdurrazaq TO, Ibikunle AA, Braimah RO (2016). Cervical necrotizing fasciitis: a potentially fatal disease with varied etiology. Ann Med Health Sci Res.

